# Critical role of growth medium for detecting drug interactions in Gram-negative bacteria that model *in vivo* responses

**DOI:** 10.1128/mbio.00159-24

**Published:** 2024-02-16

**Authors:** Kathleen P. Davis, Yoelkys Morales, Rachel J. Ende, Ryan Peters, Anne L. McCabe, Joan Mecsas, Bree B. Aldridge

**Affiliations:** 1 Department of Molecular Biology and Microbiology, Tufts University School of Medicine, & Stuart B. Levy Center for Integrated Management of Antimicrobial Resistance Boston, Boston, Massachusetts, USA; 2 Graduate School of Biomedical Sciences, Tufts University School of Medicine, Boston, Massachusetts, USA; 3 Department of Basic and Clinical Sciences, Albany College of Pharmacy and Health Sciences, Albany, New York, USA; 4 Department of Biomedical Engineering, Tufts University School of Engineering, Medford, Massachusetts, USA; Tel Aviv University, Tel Aviv, Israel

**Keywords:** antibiotic resistance, combination therapy, *Klebsiella*, *Acinetobacter*, *Pseudomonas aeruginosa*

## Abstract

The rise in infections caused by multidrug-resistant (MDR) bacteria has necessitated a variety of clinical approaches, including the use of antibiotic combinations. Here, we tested the hypothesis that drug-drug interactions vary in different media, and determined which *in vitro* models best predict drug interactions in the lungs. We systematically studied pair-wise antibiotic interactions in three different media, CAMHB, (a rich lab medium standard for antibiotic susceptibility testing), a urine mimetic medium (UMM), and a minimal medium of M9 salts supplemented with glucose and iron (M9Glu) with three Gram-negative ESKAPE pathogens, *Acinetobacter baumannii* (Ab), *Klebsiella pneumoniae* (Kp), and *Pseudomonas aeruginosa* (Pa). There were pronounced differences in responses to antibiotic combinations between the three bacterial species grown in the same medium. However, within species, PaO1 responded to drug combinations similarly when grown in all three different media, whereas Ab17978 and other Ab clinical isolates responded similarly when grown in CAMHB and M9Glu medium. By contrast, drug interactions in Kp43816, and other Kp clinical isolates poorly correlated across different media. To assess whether any of these media were predictive of antibiotic interactions against Kp in the lungs of mice, we tested three antibiotic combination pairs. *In vitro* measurements in M9Glu, but not rich medium or UMM, predicted *in vivo* outcomes. This work demonstrates that antibiotic interactions are highly variable across three Gram-negative pathogens and highlights the importance of growth medium by showing a superior correlation between *in vitro* interactions in a minimal growth medium and *in vivo* outcomes.

**IMPORTANCE:**

Drug-resistant bacterial infections are a growing concern and have only continued to increase during the SARS-CoV-2 pandemic. Though not routinely used for Gram-negative bacteria, drug combinations are sometimes used for serious infections and may become more widely used as the prevalence of extremely drug-resistant organisms increases. To date, reliable methods are not available for identifying beneficial drug combinations for a particular infection. Our study shows variability across strains in how drug interactions are impacted by growth conditions. It also demonstrates that testing drug combinations in tissue-relevant growth conditions for some strains better models what happens during infection and may better inform combination therapy selection.

## INTRODUCTION

The rise in antimicrobial-resistant (AMR) infections is a global health crisis that threatens the ability to treat many bacterial, viral, and fungal infections (1). The rate of multidrug-resistant (MDR) bacterial infections has been steadily increasing for the past 20 years and was boosted by the recent SARS-CoV2 pandemic, which saw a significant increase in MDR-related secondary bacterial infections leading to increased rates of morbidity and mortality (2). Among MDR infections, some of the most harmful and difficult to treat are those caused by the Gram-negative organisms *Klebsiella pneumoniae* (Kp), *Acinetobacter baumannii* (Ab), *Pseudomonas aeruginosa* (Pa), which along with *Enterococcus faecium*, *Staphylococcus aureus*, and *Enterobacter* species make up a group of bacteria known as ESKAPE pathogens. This group of bacteria is recognized by the World Health Organization (WHO) as capable of pan-resistance (3). Clinicians and scientists have responded by investing in antibiotic stewardship and novel therapies to combat these infections (4).

One method to treat emerging pan-resistant bacteria is the use of multiple antibiotics (5, 6). Although antibiotic combination therapy is not standard practice for most MDR infections (7), both case studies and meta-analyses have identified the benefits of combination therapy for extensively drug-resistant and pan-drug-resistant infections (5, 6, 8–10). Benefits such as improved mortality and reduction in emerging resistance are most evident in critically ill patients (5, 9, 11, 12). Despite these advantages, studies are often inconclusive in supporting combination therapy (13, 14). However, with increasing rates of infection by pan-drug-resistant bacteria, the implementation and reliance on combination therapy may also rise (15). Limitations to our understanding of drug combination therapies for the treatment of MDR infections inhibit our ability to effectively predict *in vivo* efficacy using *in vitro* assays (16). Therefore, therapy is reliant on clinical reasoning by individual physicians on a case-by-case basis. Another barrier for combination therapy testing is the resources required to test combinations of antimicrobials in a traditional plate-based checkerboard assay due to the exponential cost of testing multiple drugs in high-order combinations (17). Understanding the potential for combination therapy is further complicated by the fact that these Gram-negative bacteria can cause infections at multiple sites (3). Thus, standard-rich media conditions (as defined by the European Committee on Antimicrobial Susceptibility Testing and the International Organization for Standardization) used for *in vitro* assays may not effectively predict how individual or combinations of antibiotics will interact in various distinct *in vivo* environments where bacterial metabolism can differ (18–23).

In fact, studies have shown that growth media can impact the efficacy of antibiotics as well as reveal unsuspected targets of antibiotics (21, 23–26). To capitalize on this to improve the accuracy of antibiotic testing, some groups have started exploring antibiotic testing in host-mimicking media. These media range from supplementing synthetic media with key *in vivo* molecules such as sodium bicarbonate (27), to using human serum, urine, or synovial fluid to replicate some aspect of the infection environment (28–31). Using mimetic media can better replicate the complex interplay between the infection environment and aspects of bacterial genetics (30), physiology (31), or phenotypic variability (32) that can lead to alterations in antibiotic susceptibility. Thus, developing mimetic media systems may improve antibiotic susceptibility determination and yield insight into resistance mechanisms and bacterial population dynamics. In addition to the use of tissue mimetic media, other groups have worked to improve the accuracy of antibiotic susceptibility testing by mimicking other aspects of infection, such as the effect of reactive metabolic byproducts (33) or synergistic interactions of antibiotics with antimicrobial peptides (34–36). Here, we hypothesize that drug interactions vary by infection site growth conditions, and that antibiotic combination susceptibility measurements taken using a minimal medium containing glucose (M9Glu) will better predict *in vivo* outcomes in the lungs compared to a rich medium or a urine-mimetic medium.

To test this hypothesis, we undertook a systematic study of pairwise antibiotic interactions in different growth environments focused on three Gram-negative ESKAPE pathogens, Ab, Kp, and Pa, which together are among the major worldwide causes of nosocomial infections (3). We measured synergistic, additive, and antagonistic antibiotic interactions in standard-rich medium and compared these to measurements made in media containing nutrient sources lung or urine environments, to model two common sites of infection for these three pathogens. Finally, we assessed the capacity to translate *in vitro* measurements made using M9 salts + glucose, a carbon source that is abundant in the lungs or CAMHB to results found in a mouse lung infection model of Kp. This work demonstrates that antibiotic interactions are highly variable when comparing three Gram-negative ESKAPE pathogens and highlights the importance of growth medium by showing a superior correlation between *in vivo* interactions and *in vitro* interactions in a tissue mimetic growth medium.

## RESULTS

### Systematic survey of condition-specific drug potencies and interactions in three Gram-negative pathogens

**Fig 1:**
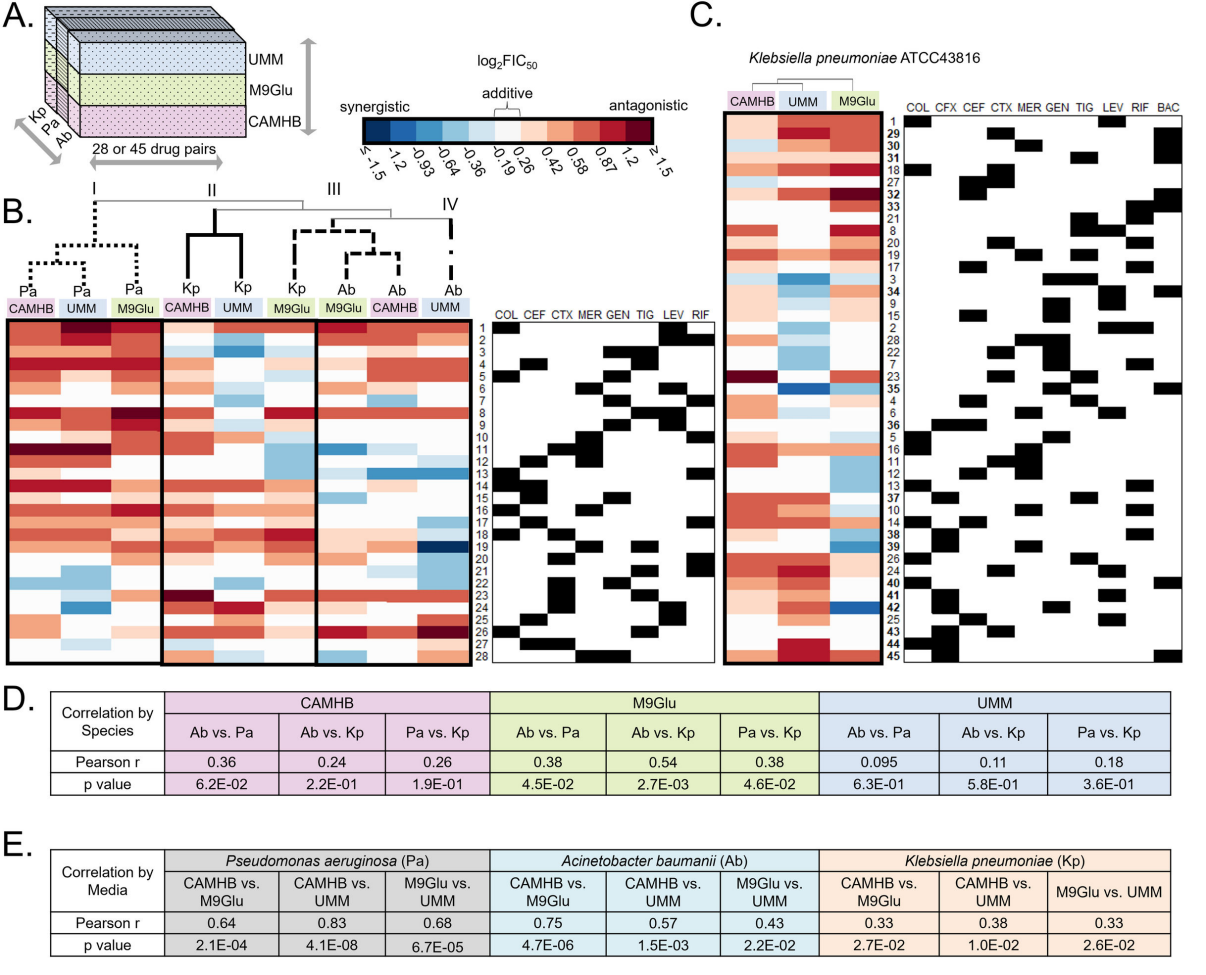
Variation in drug interaction across species and media. (**A**) Study design involved testing 28 pairs of antibiotics against *A. baumannii* ATCC17978 (Ab) and *P. aeruginosa* PaO1 (Pa), and 45 pairs against *K. pneumoniae* ATCC 43816 (Kp). Testing was done with all strains grown in three different growth conditions: cation-adjusted Mueller-Hinton Broth (CAMHB); M9 minimal medium + 0.5% glucose supplemented with 0.6 µM iron (II) sulfate, and with 10 mM sodium acetate for Ab and Pa; and urine mimetic medium (UMM) (41) supplemented with 0.01% glucose and 0.6 µM iron (II) sulfate for Kp. (**B**) Clustergram of the log_2_FIC_50_ values of the 28 drug pairs tested across all three species and media, with each row representing a drug combination (indicated by black squares under the drug abbreviations in the table on the right) and each column representing a species tested in a particular medium. Each drug-pair number is maintained throughout the manuscript for ease of comparison. Each value represents an average of at least three replicates. Hierarchical clustering was performed using average linkage between clusters and Pearson correlation distance metric between columns. Combinations tested against Pa in CAMHB, M9Glu, and UMM clustered together (Cluster I, dotted lines on tree), and combinations tested against Kp in CAMHB and UMM clustered together (Cluster II, solid lines on tree). However, combinations tested against Kp in M9Glu clustered together with combinations tested against Ab in M9Glu and CAMHB (Cluster III, dashed lines on tree), while combinations tested against Ab in UMM clustered separately (Cluster IV, dot-dash line pattern on tree). (**C**) Clustergram of the log_2_FIC_50_ values of all 45 drug pairs tested against Kp in all three media (columns). Hierarchical clustering, notation of drug pairs, and representation of log_2_FIC_50_ are the same as for (**B**). (**D**) The Pearson correlation coefficient (R) and *P* value were determined for each species-to-species comparison of mean log_2_FIC_50_ values in the three media conditions. (**E**) The Pearson correlation coefficient (R) and *P* value were determined for each medium-to-medium comparison of mean log_2_FIC_50_ values in the three species.

To determine the dependence of drug potencies and interactions on growth conditions and bacterial strains from different species, we generated a data set of single-drug potencies and pairwise drug interaction measurements from a panel of drugs that were tested against strains of Ab, Pa, and Kp (Fig. 1A). For our initial data set, we chose well-characterized strains of each species—Ab strain ATCC 17978 (Ab17978), Pa strain PaO1 (PaO1), and Kp strain ATCC 43816 (Kp43816). Each of these strains was grown in three different growth conditions (Fig. 1A) and tested against clinically relevant drugs that cover a range of classes and mechanisms of action (Table S1) (37). The drugs trimethoprim-sulfamethoxazole (BAC) and cefixime were tested only with Kp strains because of their clinical relevance specific to Kp (38). We systematically measured single-drug potencies as well as two-way drug interactions among strains and media conditions by leveraging the efficiency of a methodology called diagonal measurements of n-way drug interactions (DiaMOND), which implements a geometric optimization of the standard checkerboard assay (39, 40).

Each of these species can cause infection at multiple sites in the body, which have different growth conditions that may influence bacterial metabolism (23, 42) and drug response (20, 21, 43). However, to our knowledge, the effect of growth conditions on drug interactions across different species has not been tested systematically. To directly evaluate whether different growth conditions impact single-drug potencies and pairwise drug interactions, we employed three media conditions—Cation-Adjusted Mueller Hinton Broth (CAMHB), M9 + 0.5% Glucose + 0.6 µM Fe(II)SO4, pH 7.0 (M9Glu), and a urine mimetic medium (UMM) (41). We chose CAMHB because it is a standard for microbiological susceptibility testing, and it is a rich medium that is high in amino acids and vitamins (44). M9Glu is a simple minimal medium that lacks amino acids yet still produces consistent reproducible bacterial growth. Additionally, M9Glu reflects the high level of glucose and low amino acid availability observed in bronchoalveolar lavage fluid from mice (45–47) and humans (48) which may make it a better mimic for bacterial infection in the lungs and other amino acid deficient environments. Finally, UMM was based on Brooks and Keevil (41). UMM has a pH of 6.4 with creatinine and urea as the predominant carbon sources (see Materials and Methods) and was chosen to approximate the growth environment experienced by the bacteria during a urinary tract infection (41).

### Drug potencies and interactions are dependent on strain and growth environment

For drug potency, we measured the IC_50_, the amount of drug (μg/mL) required to achieve 50% growth inhibition. The single drug IC_50_ values for the eight drugs tested against Ab17978 and PaO1 and the 10 drugs tested against Kp43816 for each species grown in CAMHB, M9Glu, and UMM are shown in Fig. S1. Both the strain identity and the growth medium influenced drug potency. Of note, antibiotics were most potent against Pa in M9Glu with 7/8 antibiotics tested having the lowest IC_50_ values in this medium. Whereas for Ab and Kp, the potency was highest in M9Glu for 4/8 and 4/10 antibiotics, respectively. Colistin, however, was the most potent in CAMHB for all three strains. We next compared the fold-change in IC_50_ within each medium for each strain and antibiotic. Only 15 IC_50_ values increased by fivefold or more when compared to the most potent medium for each strain and antibiotic condition, and all 15 were statistically significant. Eleven of the 15 fivefold or more increases in IC_50_ values occurred in UMM; however, only levofloxacin required more than fivefold the amount of antibiotic in UMM for all three strains. Finally, Pa had the greatest number of conditions with at least a fivefold change in drug potency (7/16); while Ab (4/16) and Kp (4/20) had the lowest. Thus, among these bacterial strains, Kp43816 exhibited the least variation in single-drug potencies across different growth conditions.

Next, we generated a drug interaction data set using DiaMOND (39, 49) by measuring the three most information-rich dose–response curves: the combination dose responses of increasing equipotent doses of two drugs, and the dose responses of every single drug. We used these dose–response curves to calculate the fractional inhibitory concentration (FIC), a measure of drug interactions. The FIC is the ratio of the observed combination dose that results in a certain level of growth inhibition compared to the expected combination dose if the two drugs are additive (see Materials and Methods). Here, we report log transformed FIC scores; log_2_FIC_50_ scores close to 0 indicate additivity, more negative scores indicate synergy (e.g., the drugs combined are more effective than expected based on their efficacies alone), and more positive scores indicate antagonism between drug pairs. The efficiency of DiaMOND enabled us to create a data set of >300 unique combinations of species, medium, and pairwise drug interactions.

The drug interaction data for 28 drug pairs tested for Ab17978, Kp43816, and PaO1, each grown in three media conditions, is shown in a heatmap with hierarchical clustering in Fig. 1B. We observed that for each medium (color-coded above the clustergram), drug combinations varied in their log_2_FIC_50_ scores among the different strains. Furthermore, within individual strains, drug combinations often varied in their log_2_FIC_50_ scores across the three media conditions (three columns within a black box), although the extent of this variation is different for different strains. The three PaO1 growth conditions cluster together (Cluster I), indicating their similarity to each other and differences from Ab17978 and Kp43816. On the other hand, Ab17978 CAMHB and M9Glu cluster together with Kp43816 M9Glu (Cluster III), while Ab17978 UMM is in an adjacent cluster (Cluster IV). Kp43816 CAMHB and Kp43816 UMM make up Cluster II. These clustering patterns suggest that drug interactions are influenced by differences between strains while the impact of media is more pronounced in some strains versus others.

Pearson correlation coefficients were derived to quantify changes in drug interactions between different strains in the same growth conditions (Fig. 1D) and between different growth conditions within each strain (Fig. 1E). The outcome of drug pair interactions between strains within the same medium was extremely variable; all nine Pearson coefficients were below 0.6 and eight of the nine were below 0.4 (Fig. 1D). Thus, strain-specific attributes impact drug interactions when strains are grown in the same medium. Curiously, despite the overall low Pearson coefficients, the correlation between strains was consistently highest in M9Glu (*r* = 0.38–0.54) and lowest in UMM (*r* = 0.095–0.18) (Fig. 1D). In contrast to differences in drug interactions between strains grown in the same medium, drug interactions in PaO1 between all three media showed high and significant correlations, with all three correlations above 0.64 (Fig. 1E). This was also reflected visually by the clustergrams (Fig. 1B). Likewise, drug interactions in Ab17978 between CAMHB and M9Glu showed a high and significant correlation (Fig. 1E). On the other hand, Kp43816 had low correlations in medium-to-medium comparisons, with all three correlations below 0.4 (Fig. 1E). In summary, drug interactions varied widely across different strains, while media conditions had larger effects on drug interactions in some strains compared to others. This contrasts with the single-drug potency data, in which the most variation in drug potencies across different growth conditions was observed for PaO1, and the least was observed for Kp43816.

### Drug interactions are overall biased toward antagonism, but synergy is more prevalent in some strains in nutrient-limited media

Efforts to develop clinically impactful combination therapies are focused on identifying synergistic combinations. Though we did not find combinations that were synergistic across all strains and conditions tested, one combination, ceftriaxone + gentamicin (#22) was synergistic in a single medium (UMM) across all three strains tested. Additionally, two combinations, colistin + rifampicin (#13) in Ab17978 and gentamicin + tigecycline (#3) in Kp43816, were synergistic across all three growth conditions. However, combinations that were synergistic against one species in a particular growth condition were often not synergistic against other species in that growth condition nor in a different growth condition for the same species [e.g., meropenem plus tigecycline (#19) was synergistic for Ab17978 in UMM, but antagonistic for Ab17978 in the other growth conditions, and antagonistic for PaO1 and Kp43816 in UMM]. One combination was antagonistic across all species and media, colistin + levofloxacin (#1). The tendency towards antagonism was dependent on growth conditions, with combinations in UMM less likely to be antagonistic than those in CAMHB or M9Glu. Specifically, nine combinations were antagonistic across all three species in CAMHB (#1, #4, #5, #6, #8, #14, #18, #19, and #26), eight in M9Glu (#1, #4, #8, #18, #19, #20, #23, and #26), and one in UMM (#1) (Fig. 1B).

**Fig 2:**
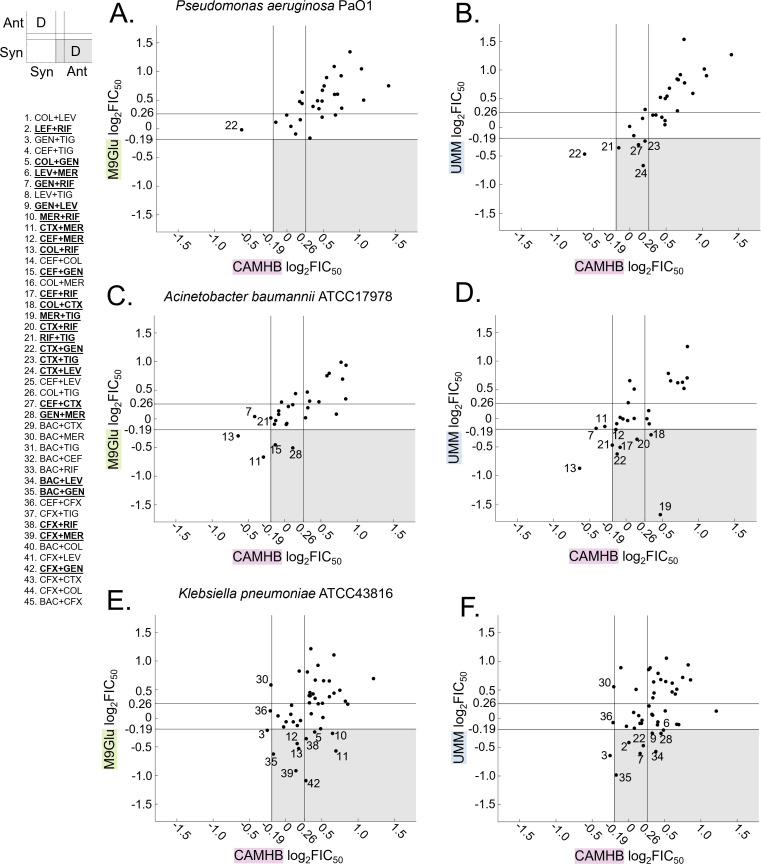
Nutrient-limited media (M9Glu, UMM) reveal more synergistic combinations than standard-rich media (CAMHB) for *Klebsiella pneumoniae*. (**A–F**) Scatterplots of log_2_FIC_50_ values for all 28 drug pairs tested against (**A, B**) *Pa* PaO1, (**C, D**) *Ab* ATCC17978 and (**E, F**) 45 drug pairs tested against *Kp* ATCC43816. *X*-values represent log_2_FIC_50_ in CAMHB, while *y*-values represent log_2_FIC_50_ value in nutrient-limited media, (**A, C, E**) M9Glu and (**B, D, F**) UMM. Lines parallel to the *x*-axis and *y*-axis indicate the boundaries of additivity (log_2_FIC_50_ from −0.19 to 0.26, see Materials and Methods). Combinations that fall in the upper-left and lower-right sections of each graph indicate discordant interactions between results in CAMHB and results in the nutrient-limited medium (marked with a (D) in the key on the left). Combinations that fall in the gray-shaded regions are synergistic in nutrient-limited media but additive or antagonistic in CAMHB; combinations for which this occurs in one or more species are bold-faced and underlined in the list of combinations on the left.

Despite the overall predominance of antagonism, the number of combinations that were additive or antagonistic in CAMHB but synergistic in one or both nutrient-limited media differed for the three species (Fig. 2, gray regions of graphs). For PaO1, 27 combinations were additive or antagonistic in CAMHB, while only one was synergistic. We noted the number of times that combinations switched from additivity or antagonism in CAMHB to synergy in either M9Glu and/or UMM. Out of the 54 chances for a switch, only four combinations switched to synergy (all in UMM); thus, 4.7% of all possible switches occurred in PaO1. For Ab17978, more combinations shifted from additive or antagonistic in CAMHB to synergistic in a nutrient-limited media: two synergies were identified in M9Glu (Fig. 2C, gray region) and six synergies were found in UMM (Fig. 2D, gray region), for a total of eight switches to synergy. Since there were 24 combinations that were additive or antagonistic in CAMHB that could switch to synergy in M9Glu and/or UMM, 16.7% of all possible switches occurred in Ab17978. For Kp43816, among the drug pairs tested in all three species, five combinations were synergistic in M9Glu but not in CAMHB (Fig. 2E, gray region), and six combinations were synergistic in UMM but not in CAMHB (Fig. 2F, gray region). Among the drug pairs tested only in Kp43816 (e.g., the combinations including trimethoprim-sulfamethoxazole or cefixime)*,* four were synergistic in M9Glu but not CAMHB, and two were synergistic in UMM but not CAMHB. For Kp43816, 51 of the 54 total combinations tested were additive or antagonistic in CAMHB, and a total of 17 switched to synergy in UMM and/or M9Glu (16.7% of all possible switches). Thus, for Ab17978 and Kp43816, nutrient-limited conditions revealed additional synergistic combinations, while for the strain of Pa we tested (PaO1), testing drug pairs in rich media sufficient.

### Specific antibiotics were associated with synergistic interactions in nutrient-limited media

**Fig 3:**
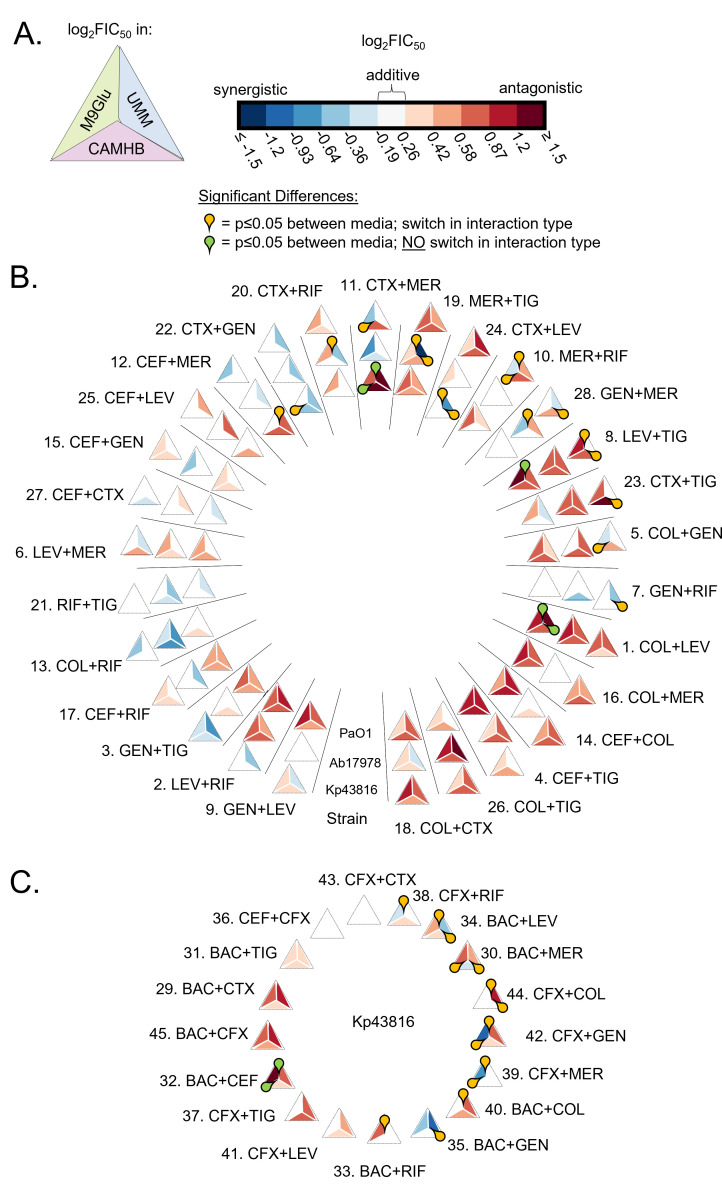
Different strains show different degrees of variation across media conditions. (**A**) Triangle diagram represents how the log_2_FIC_50_ data are depicted in (**B**) and (**C**) with UMM value on the top right, M9glu value on the top left, and CAMHB on the bottom. Log_2_FIC_50_ values are reported as in Fig. 1. (**B, C**) Yellow teardrops indicate significant differences (*P* ≤ 0.05) between media where combinations change interaction type (e.g., switch from synergy to antagonism between media); green teardrops indicate significance for combinations that do not change interaction type. Significance was based on a two-way ANOVA using Tukey’s multiple comparison post-test (*α* = 0.05), using the log_2_FIC_50_. (**B**) The innermost ring of triangles represents log_2_FIC_50_ data of combinations tested in Pa, the second ring from the middle represents combinations tested in Ab, and the outermost ring represents the combinations tested in Kp. (**C**) The log_2_FIC_50_ data for combinations only tested against Kp.

We next evaluated if specific antibiotics were more likely to be impacted by changes in media and if certain drugs were responsible for higher rates of synergistic interactions dependent on growth medium. To investigate this, we first determined which combinations showed significant differences in log_2_FIC_50_ scores in different media conditions with the same species. The results are shown in Fig. 3 with statistically significant differences between combinations indicated with a teardrop. These data are the same as in Fig. 1 but are replotted in this format to highlight trends in media-dependence in a species and drug-specific manner.

First, we considered the 28 drug pairs that we tested in all three strains (Fig. 3B). For PaO1 (Fig. 3B, innermost ring), there were nine instances of significant differences between interaction measurements in two media, but in five of those cases, the interaction type did not change between the two media (green teardrops). For Ab17978 (Fig. 3B, second ring from the middle), there were four instances of significant differences between interaction measurements, and in all four cases the type of interaction (synergy, additivity, and antagonism) for a combination switched between two media (yellow teardrops). Finally, for Kp43816 (Fig. 3B, outermost ring), there were nine instances of significant differences, and in all cases the interaction type switched (yellow teardrops). Among the additional combinations tested in Kp43816 (Fig. 3C), we saw sixteen significant differences, and the interaction type changed for 14 of those cases (yellow teardrops) and stayed antagonistic for two cases (green teardrops). Thus, significantly different interaction type switches between media were more frequent in Kp43816 than Ab17978 or PaO1*,* mirroring the same trend observed with the Pearson correlation coefficients where there was the poorest correlation for Kp43816 (Fig. 1E).

**Fig 4:**
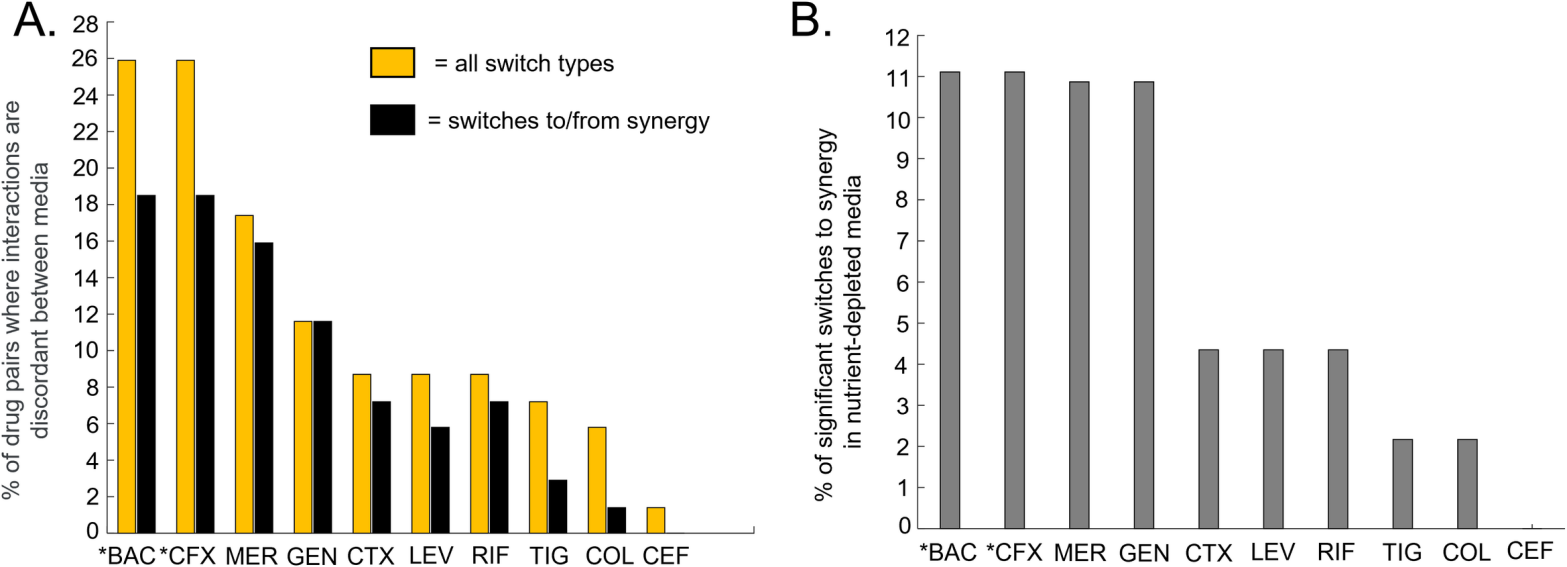
Some drugs were more frequently observed in combinations that show a significant difference in interaction between media. An asterisk indicates a drug that was only tested against Kp. (**A**) Yellow bars: the percentage of combinations involving each drug that showed a statistically significant log_2_FIC_50_ interaction type switch (e.g., synergistic to antagonistic) in different media conditions (yellow bars). Black bars: the percentage of combinations involving each drug that showed a statistically significant log_2_FIC_50_ interaction type switch to or from synergy. (**B**) The percentage of combinations involving each drug that switched from additivity or antagonism in CAMHB to synergy in nutrient-limited media (M9Glu or UMM).

Next, we evaluated whether some drugs were over-represented among significantly different combinations that had instances of switching interaction type among media (e.g., additive to antagonistic or synergistic to antagonistic, Fig. 3, yellow teardrops). Because two drugs in the data set (trimethoprim-sulfamethoxazole and cefixime) were not tested against Ab17978 or PaO1*,* we converted the actual number of interaction switches to a percentage of the total possible interaction switches between media types, for combinations containing that drug. The total number of possible interaction switches was 27 for trimethoprim-sulfamethoxazole and cefixime and 69 for the other eight drugs (see Materials and Methods). The results for all 10 drugs are shown in Fig. 4A (yellow bars). Combinations involving trimethoprim-sulfamethoxazole, cefixime, meropenem, and gentamicin were more likely to show a significant difference in interaction type switch between media. We also calculated what subset of the instances of switching involved synergy—i.e., they were not a switch from additivity to antagonism (Fig. 4A, black bars). Of these, over 80% of the switches with gentamicin and meropenem involved a change to or from synergy as indicated by the minimal differences between black bars and yellow bars (Fig. 4A).

We determined the subset of instances that involved switching from additivity or antagonism in CAMHB to synergy in a nutrient-limited media for each antibiotic (Fig. 3B and C, yellow teardrops). To do so, the number of switches to synergy in nutrient-limited media was divided by the total number of possible switches between CAMHB and M9Glu or UMM. Trimethoprim-sulfamethoxazole and cefixime had the highest percentage of significant switches to synergy in a nutrient-limited media (Fig. 4B), with the caveat that they were not tested in Ab17978 or PaO1. Of the eight drugs tested in all three strains, meropenem and gentamicin had the highest percentage of significant switches to synergy in nutrient-limited media (five switches for meropenem and five for gentamicin). Thus, nutrient-limited media revealed synergies not observed in CAMHB as exemplified with combinations that included meropenem and gentamicin, as well as with trimethoprim-sulfamethoxazole and cefixime in Kp43816.

The tendency for combinations involving meropenem and/or gentamicin to be synergistic did not relate to any obvious patterns in gentamicin and meropenem potencies. While gentamicin potency showed a lot of variation between media types for all three strains [eight out of nine medium-to-medium comparisons across all three strains were significantly different with three of these requiring more than fivefold more drug to reach equal potency (Fig. S1)], meropenem had less variation (only six out of nine comparisons were significantly different with two requiring more than fivefold more drug to reach equal potency). Furthermore, levofloxacin was the only other drug for which eight out of nine medium-to-medium comparisons across all three species were statistically significantly different, but it had a relatively low percentage of significant switches to synergy in nutrient-limited media (Fig. 4B). Overall, trends in single-drug potency do not appear to be particularly informative of drug interactions containing those drugs, thus highlighting the importance of strain-specific combination testing in relevant growth conditions. Our *in vitro* data indicate that growth conditions influence the likelihood of observing synergistic combinations, with the dependency on growth condition being strongest for Ab17978 and Kp43816 (Fig. 2 to 4A), and that certain antibiotics are more likely to impact medium-dependent synergies.

### Ab and Kp clinical isolates recapitulate media effects observed for Ab17978 and Kp43816

Next, we sought to determine if the trends observed for Ab17978 and Kp43816 regarding correlation (for Ab17978) or lack thereof (for Kp43816) between drug interaction in M9Glu versus CAMHB held for other clinical isolates of Ab and Kp. To do this, three Ab clinical isolates (Ab5075, EGA355, and EGA368) and four Kp clinical isolates (UCI38, MGH47, BWH15, and BIDMC33B) were grown in CAMHB and M9Glu and tested with different antibiotics combinations.

**Fig 5:**
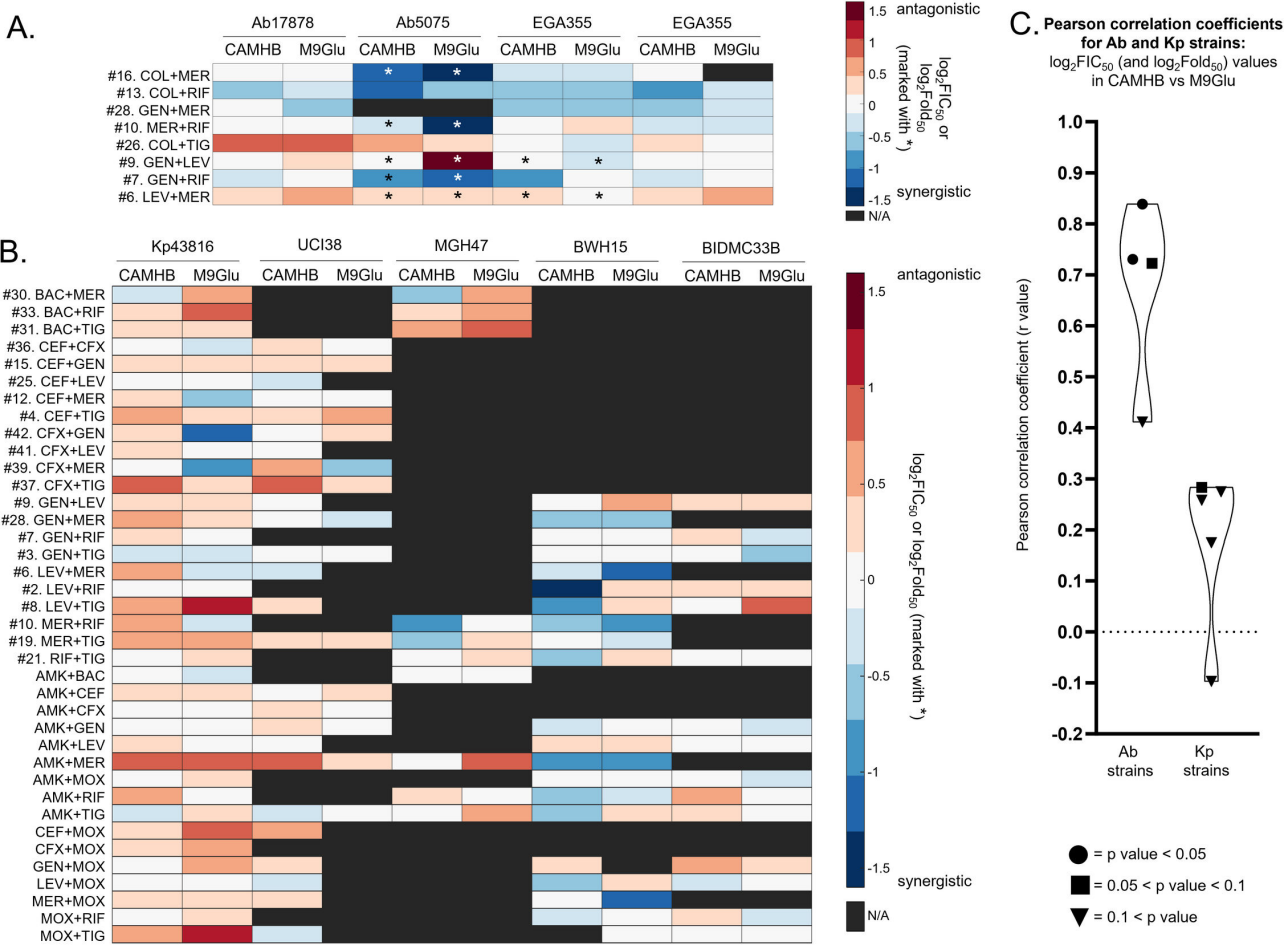
Media effects observed for Ab17978 and Kp43816 are recapitulated by Ab and Kp clinical isolates. (**A**) log_2_FIC_50_ and log_2_Fold_50_ values for combinations tested against Ab clinical isolates (Ab5075, EGA355, and EGA368) and lab strain (Ab17978) grown in CAMHB (left) and M9Glu (right). The log_2_Fold_50_ values are indicated with an asterisk. (**B**) log_2_FIC_50_ values for combinations tested against Kp clinical isolates (BIDMC33B, BWH15, UCI38, and MGH47) and lab strain (Kp43816) grown in CAMHB (left) and M9Glu (right). For (**A**) and (**B**), not all combinations were tested against all strains, due to variation in isolate resistance profiles; combinations not tested are shown in black. All values are averages of at least three biological replicates. (**C**) Pearson correlation coefficients (*r* values) for comparing log_2_FIC_50_ values in CAMHB versus M9Glu (shown in (A)) for Ab17978 and Ab clinical isolates (left violin), and Pearson correlation coefficients (*r* values) for comparing log_2_FIC_50_ values in CAMHB versus M9Glu (shown in (B)) for Kp43816 and Kp clinical isolates (right violin).

The Ab clinical isolate Ab5075 was highly resistant to gentamicin and meropenem, while the EGA355 isolate was highly resistant to levofloxacin resulting in unobtainable IC_50_ values for these drugs (Fig. S4A). Thus, for these drugs, we tested for potentiation in the relevant strain by adding a constant amount of the resistant drug along with increasing amounts of the sensitive drug and measuring shifts in IC_50_ of the sensitive drug. This shift was reported as a log_2_ fold-change in IC_50_, with negative log_2_Fold_50_ scores indicating that the addition of the resistant drug lowered the IC_50_ of the sensitive drug, despite the resistant drug showing no growth inhibition on its own (Fig. 5A). The three Ab clinical isolates recapitulated the trend observed for Ab17978 of high Pearson correlation coefficients when comparing log_2_FIC_50_ (and log_2_Fold_50_) values from growth in CAMHB versus M9Glu. For the combinations shown in Fig. 5A, the Pearson correlation coefficients (Fig. 5C) were 0.73, 0.72, 0.41, and 0.84 for Ab17978, Ab5075, EGA355, and EGA368, respectively. We note that not all of these correlations achieved statistical significance (*P* values were 0.040, 0.067, 0.31, and 0.018, respectively), likely due to the fewer number of combinations tested (compared to the 28 tested against Ab17978).

Due to the variable antibiotic resistances of the Kp strains (Fig S4B), only combinations where the strains were sensitive to both drugs were tested and only log_2_FIC_50_ values were measured (Fig. 5B). Additionally, combinations with amikacin (AMK) and moxifloxacin (MOX) were added due to the clinical relevance of these drugs. In contrast to the Ab strains, the Pearson correlation coefficients for the Kp strains were uniformly low (Fig. 5C), with values of 0.28, 0.18, 0.27, –0.10, and 0.26 for Kp43816, UCI38, MGH47, BWH15, and BIDMC33B, respectively (*P* values of 0.085, 0.53, 0.44, 0.69, and 0.35), indicating poor correlation of antibiotic combination behavior between growth in CAMHB and M9Glu for both Kp43816 and Kp clinical isolates. Taken together, these data suggest that combination drug responses in CAMHB versus M9Glu for Ab17978 and Kp43816 are likely not a unique feature of those two strains and may be present more broadly among Kp and Ab isolates.

### Drug combination outcomes in a Kp mouse lung infection model were better predicted by *in vitro* measurements in M9Glu

To evaluate the ability of *in vitro* media conditions to predict the efficacy of a drug combination *in vivo,* we adapted a mouse model for Kp lung infection to incorporate antibiotic therapy (45, 46, 50, 51). Though traditional drug therapy is designed with the goal of eliminating bacterial burden, we used subtherapeutic doses of antibiotics with the goal of capturing potential synergies in the treatment of tissue infection by Kp. Specifically, we sought doses of single antibiotics (monotherapy) that reduced the lung bacterial burden significantly compared to a vehicle control, but where the bacterial burden remained at detectable levels. If combination treatments were more effective, we would expect fewer colony-forming units (CFUs) recovered versus the single doses.

**Fig 6:**
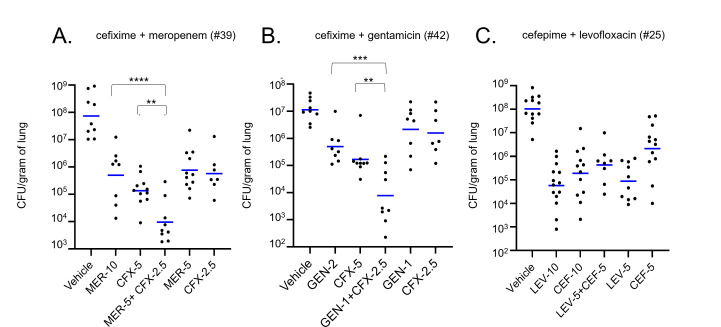
Drug combinations identified as synergistic in M9Glu, but not CAMHB, significantly reduce lung bacterial burden during mouse lung infection by *Klebsiella pneumoniae*. (**A–C**) Swiss Webster wild-type mice (black circles) were infected via intranasal route with 10,000 CFUs of Kp43816 and infection was allowed to proceed for 14 h at which point mice were treated with either DMSO or indicated doses of drugs (in mg/kg) via intraperitoneal injection. Mice receiving meropenem were given a second dose at 18 h due to its short *in vivo* half-life (52). Lungs were harvested after 22 h post infection and plated for bacterial burden (CFU/g of lung). Blue lines indicate geometric means. Data for each drug combination group were compiled from *n* = 3 independent experiments with three to four mice in each group. Statistical analysis was done by two-way ANOVA with Bonferroni corrections.

We tested the hypothesis that M9Glu is better able to predict *in vivo* outcomes by testing two combinations of antibiotics that were synergistic in M9Glu, cefixime + meropenem (#39) and cefixime + gentamicin (#42), but additive or antagonistic, respectively, in CAMHB. These combinations were chosen because they were statistically significant *in vitro* (Fig. 3). Initial testing was done to identify roughly equipotent doses that met the criteria for subtherapeutic doses. Doses of 10 mg/kg of meropenem, 5 mg/kg of cefixime, and 2 mg/kg of gentamicin given at 14 h post-infection resulted in a lung bacterial burden between 10^5^ and 10^6^ CFUs 22 h post-infection after intranasal inoculation of 10,000 CFUs, whereas untreated controls ranged between 10^7^ and 10^8^ CFUs. This bacterial burden in treated mice was significantly lower than the non-treated vehicle control while still being 2–3 logs higher than the limit of detection for this assay (Fig. 6).

To translate the additivity model used in DiaMOND and compare drug combination therapies to monotherapies *in vivo*, roughly equipotent doses of antibiotics were used for monotherapies and compared to combinations of two drugs, each used at half the equipotent dose. For example, 5 mg/kg of meropenem + 2.5 mg/kg cefixime was compared to 10 mg/kg meropenem or 5 mg/kg cefixime. When either cefixime + meropenem (#39) or cefixime + gentamicin (#42) was used to treat Kp-infected mice, the combination therapy significantly reduced lung bacterial burden compared to their respective monotherapies (Fig. 6A and B). Including the individual components of the antibiotic combination doses on their own allowed for quantifying drug interaction via a modified Bliss independence score (53) using log_10_-transformed values for CFU (see Materials and Methods). In brief, the Bliss independence model compares the observed effect of the combination to an expected inhibitory effect of the combination which assumes the two drugs act independently; positive scores indicate synergistic interactions, while negative Bliss scores indicate antagonistic interactions. Using this log-transformed Bliss independence statistic, synergistic Bliss interactions scores of 0.11 ± 0.02 for cefixime + meropenem (#39) and 0.24 ± 0.07 for cefixime + gentamicin (#42) were calculated. Taken together, the significant reduction in lung bacterial burden by the combinations in addition to the positive Bliss scores indicate that these two combinations were acting synergistically in the mouse lung. Therefore, the drug interactions *in vivo* are more closely correlated with the *in vitro* measurements in M9Glu rather than the additive or antagonistic interactions measured in CAMHB.

To evaluate whether combination therapies broadly acted more effectively than monotherapies in this model regardless of drug interactions measured *in vitro*, cefepime + levofloxacin (#25) was used for *in vivo* testing—a combination that acted additively in both M9Glu and CAMHB. Doses of 10 mg/kg for both drugs were chosen for monotherapy and 5 mg/kg of each for combination therapy. The combination of cefepime + levofloxacin (#25) was not significantly different from either monotherapy alone and had an antagonistic log transformed Bliss interaction score of −0.22 ± 0.07 (Fig. 6C). Although the *in vivo* behavior of the cefepime + levofloxacin was slightly discordant compared to the additive interaction predicted *in vitro*, this combination therapy was not more broadly effective than monotherapy in this model. Taken together, these experiments demonstrate that by using subtherapeutic antibiotic doses, the mouse model resolved differences in single versus combination drug therapy. Additionally, these data demonstrate that for both the combinations of cefixime + meropenem (#39) and cefixime + gentamicin (#42) M9Glu medium, an *in vitro* medium more nutritionally restricted than CAMHB, was better able to predict *in vivo* behavior.

## DISCUSSION

Our results demonstrate that drug interactions differ considerably across strains. Within strains, drug interactions also vary among growth conditions. Furthermore, among the panel of drug combinations tested, we observed little to no correlation between three representative strains from different Gram-negative species, in any of the media tested (Fig. 1D) and no drug combination was synergistic across all strains and growth conditions. This discordance in response to drug combinations across different strains raises the important and unanswered question of how to best assess combination therapies. Our observation that one cannot necessarily extrapolate from one bacterial strain to another from a different species has also been reported by other investigators. Brochado et al. tested pairwise combinations from a broad array of antibiotic classes against *Escherichia coli*, *S. Typhimurium* and Pa grown in Lysogeny Broth, and found that more than 70% of their tested drug interactions were species-specific (51). This variation in response to antibiotic combinations amongst strains from different species could be due to differences in antibiotic uptake (54, 55), cell wall permeability (56), and/or cellular processes when grown in complex nutrient environments (57). Drug interactions may be dependent on media for a variety of reasons, including differences in metabolic state (58, 59), the activity of efflux pumps (60, 61), and stress response pathways which can change depending on media condition (62, 63). Consistent with the latter idea is our observation that differences in drug interactions between strains from different species were the least evident in the simplest medium, M9Glu (Fig. 1D). Collectively, our findings indicate that informed use of combination therapies should take account of species and infection sites. Further, for some species, growth conditions may have an outsized effect on combination interactions, as we have started to observe with this work and that there may not be a “golden” combination that will be synergistic across a range of species and infection sites. Given the potential impact of species-specific physiology on drug interactions, a more tailored strategy focused on the pathogen and sites of infection may need to be considered. For multi-site infections, choosing a combination that performs well across a range of growth conditions might be the best strategy.

Among our systematic drug interaction measurements, antagonism was overall more frequent than synergy (Fig. 1B and C), which is in agreement with studies of other species (40, 51, 64–67) as well as with cancer therapies (68). However, ceftriaxone + gentamicin was synergistic across strains from three different species (Ab17978, PaO1, and Kp43816) tested in UMM (Fig. 1B). There are other *in vitro* and clinical evidence of synergy for combinations of beta-lactams and aminoglycosides in both Gram-positive and Gram-negative bacteria. For example, synergy was observed in the more rapid clearance of *S. aureus* from cardiac vegetations in a rabbit endocarditis model by penicillin combined with gentamicin (69); a similar effect was also observed with *Streptococcus sanguis* in the rabbit endocarditis model (70). Synergy was also observed with amoxicillin in combination with gentamicin when used to treat various strains of *Streptococcus pneumoniae* in a mouse pneumonia model that varied in their penicillin susceptibility (71). In these cases, the cephalosporin is believed to weaken the cell wall allowing better penetration of the aminoglycoside (72–74). Some *in vitro* studies with Pa have shown synergy with a beta-lactam and aminoglycoside (75), but in the case of Pa, synergy appears to depend on the strain as well as the specific identity of the beta-lactam with an aminoglycoside in combination (76, 77). These observations and the synergy of ceftriaxone + gentamicin against all three pathogens tested in UMM further support the idea that beta-lactam + aminoglycoside antibiotic combinations may be particularly beneficial for the treatment of complex urinary tract infections caused by MDR bacteria.

Our data set allowed us to take an in-depth look at how drug interactions vary across growth conditions and in strains from different species. Though we focused on statistically significant interaction differences (Fig. 3 and 4), we reported all media-to-media interaction differences (Fig. 1) for consideration. For Kp43816 and Ab17978, combinations that included gentamicin or meropenem were more likely to change to synergistic when moving from a rich medium (CAMHB) to non-rich media (M9Glu or UMM) (Fig. 4B). This highlights the importance of testing combinations involving these drugs in non-rich growth conditions which may better reflect *in vivo* outcomes for some types of Kp and Ab infections. However, this trend with gentamicin and meropenem was not observed in PaO1. The relatively low discordance in drug interaction across media for PaO1 may be explained by Pa metabolic adaptability, minimal nutritional requirements, and ability to grow in a variety of different environments (59). These features combined with a wide array of innate resistance mechanisms (78) suggest that Pa may be able to face challenges from multiple antibiotics concurrently, along with environmental stressors. In contrast, Kp undergoes shifts in metabolism upon growth in glucose or other changes in carbon sources (79, 80), and exposure to subinhibitory amounts of meropenem also shifts the metabolism of Kp (81). It would stand to reason that a reverse of this also occurs, that changes in Kp metabolism will exert an effect on drug interaction.

The finding that measurements in M9Glu predict *in vivo* efficacy for Kp43816 is consistent with several previous studies examining the efficacy of drug combinations for Kp. One study conducted by Hirsh et al. used a neutropenic model of pneumonia for Kp to test several combinations of antibiotics for their effectiveness (82). Interestingly they found that the combination of amikacin + doripenem was effective at reducing lung bacterial burden. In our study, the combination of gentamicin + meropenem (#28), which are in the same classes as amikacin + doripenem respectively and we considered to be comparable, was found to be additive in M9Glu but antagonistic in CAMHB (Fig. 1B and C). Additional studies using neutropenic pneumonia models found that the combination of an aminoglycoside and a cephalosporin is effective at reducing lung bacterial burden (83, 84). One such combination in our study, ceftriaxone + gentamicin (#22) was additive in both CAMHB and M9Glu (Fig. 1B and C). However, another aminoglycoside + cephalosporin combination, cefixime + gentamicin (#42), was synergistic in M9Glu, but antagonistic in CAMHB (Fig. 1B and C). Altogether, these previous *in vivo* studies are in closer alignment with M9Glu than CAMHB, suggesting that it is a better medium to predict the *in vivo* efficacy of drug interactions against Kp43816.

Several studies identified effective combinations of antibiotics in *in vivo* using models of pneumonia with Ab (85–87). Taken together, these *in vivo* studies identified four effective drug combinations. All four comparable combinations tested in our study were additive or synergistic when measured against Ab strains in M9Glu, with the exception of meropenem + rifampicin (#10) against EGA355 (Fig. 5B). Further, other studies that utilize Ab in models of pneumonia identified four combinations as ineffective (86–89). Of these four combinations, all four were additive or antagonistic against Ab17978 and EGA358 in M9Glu, and three of the four were additive or antagonistic against Ab5075 in M9Glu (Fig. 5B). Overall, for both Ab and Kp, drug interaction measurements taken in M9Glu were largely in agreement with previous *in vivo* models of pneumonia and for Kp43816, more in agreement than those in CAMHB.

Collectively, these analyses indicate that for Kp, M9Glu is better able to predict *in vivo* outcomes in the lungs when compared to CAMHB (or UMM). This further implies that Kp is using a glycolytic program during its growth in the lungs and that these drug combinations are more effective under these conditions. Additionally, our results for cefixime + meropenem (#39) align with previous clinical trial results, further supporting the efficacy of double beta-lactam therapy for multidrug-resistant Kp (90). Though our results show that M9Glu is a more predictive model of *in vivo* outcomes in the lung for Kp43816, we recognize that M9Glu is not an exact mimetic of lung conditions. For example, lungs contain detectable, albeit low, and insufficient levels of amino acids for the growth of Kp autotrophs, but there are no amino acids in M9Glu (46). In addition to amino acids, there are other differences between the lungs and M9Glu. For instance, the pH of healthy lungs is in the range of 7.3–7.4 (91), while our medium was pH 7. Our lung mimetic medium had iron and glucose, but we did not supply other trace metals or additional carbon sources (59) which can impact bacterial physiology and/or antibiotics. The addition of mucus has been used in several *in vitro* models and can impact the growth of bacteria and the effectiveness of antibiotics (92), but was not included in our medium. Finally, studies have shown that cell culture medium can also be a good predictor of lung environments, based on transcriptional analysis and chemical composition of the media (24, 25, 55, 93). Future experiments may reveal that changes to the composition of M9Glu would yield a growth medium with better predictive power *in vivo*.

Traditional drug therapy in mice is often designed with the goal of eliminating the bacterial burden by utilizing full doses of each drug together. Our dosing strategy, which uses combinations with half the dose of the monotherapy, was designed to measure *in vivo* drug interactions relative to additivity as a null model (94, 95). This allowed for a more direct comparison between a combination and its respective monotherapies. A potential strength of using subtherapeutic concentrations is the resolution to detect both decreases and increases in bacterial burden when treated with a combination of drugs. Although we weighed our drug doses to detect further decreases in bacterial burden when using combinations, this model can be optimized to better capture antagonistic interactions by raising both the doses. Additionally, this dosing strategy using subtherapeutic concentrations can be adapted to test whether other infection site-specific mimetic media can achieve the same recapitulation observed here. If so, then not only could tissue mimetic media be used to better predict *in vivo* outcomes in corresponding infection sites, but the results of a panel of tissue mimetic media could be used to identify combinations that perform well across multiple sites in more complex infections. Overall, our study highlights the need for more studies to further characterize the effect of strain, species, and growth conditions on drug interactions, to inform the design of better combination therapy.

## MATERIALS AND METHODS

### Strains, antibiotics, and growth conditions

Strains used in this paper include Ab ATCC 17978 (a generous gift from the lab of Ralph Isberg at Tufts University), Pa PaO1 (a generous gift from the lab of Paul Blainey at the Broad Institute), and Kp ATCC 43816 (purchased from ATCC), as well as three Ab clinical isolates and four Kp clinical strains. Ab5075 is a well-characterized, extensively drug-resistant (XDR) isolate from a Walter Reed Army Medical Center patient between 2008 and 2009 (96–98). Susceptibility and resistance information for this strain was obtained from (96, 99). EGA355 and EGA368 (obtained from Eddie Geisinger, Northwestern University) are two Ab strains that were isolated from patient sputum samples in 2013 and 2014, respectively, by the Tufts Medical Center Microbiology Laboratory. Ab clinical isolate species confirmation and MLST strain type (ST2) were determined by whole-genome sequencing. The Kp clinical isolates UCI38, MGH47, BWH15, and BIDMC33B (obtained from the lab of Dr. Deborah Hung at the Broad Institute) were collected from urine, wound fluid, a peritoneal sample, and respiratory sample, respectively (100).

Twelve antibiotics were used in this study. Cefepime, colistin, ceftriaxone, gentamicin, levofloxacin, trimethoprim, sulfamethoxazole, cefixime, meropenem, amikacin and moxifloxacin were obtained from Sigma. Rifampicin and tigecycline were obtained from T.C.I. Chemicals. For *in vitro* studies trimethoprim and sulfamethoxazole were mixed at a 1:20 ratio. Cation-Adjusted Mueller Hinton II Broth (CAMHB) was purchased from Becton-Dickinson (BBL, Sparks, MD, USA) and prepared according to the manufacturer’s instructions. M9 Minimal Salts 5× was purchased from Becton-Dickinson (Difco, Sparks, MD, USA), and M9 Minimal Medium (M9Glu) was prepared according to the manufacturer’s instructions (including addition of 0.5% glucose). M9 was supplemented with 0.6 µM Fe(II)SO_4_ for growing all strains, and with 10 mM NaC_2_H_3_O_2_ for growing Ab and Pa strains. UMM was prepared according to the recipe of Brooks and Keevil (41) and supplemented with 0.6 µM Fe(II)SO_4_ and 0.01% glucose when used for growing Kp ATCC 43816.

### Drug potency and drug interaction measurement with DiaMOND assays

First dose centering experiments were performed to determine the IC_90_ values of each antibiotic for each strain in each medium. The same experimental protocol was used for both DiaMOND and dose centering experiments: a culture was grown overnight to saturation in the medium to be tested at 37°C with shaking, then 6 µL of culture was used to inoculate 3 mL fresh media, and this day culture was grown at 37°C with shaking until it reached mid-log (OD_600_ = 0.2–0.5). This day culture was then diluted to OD_600_ = 0.001, and 50 µL culture was added to each of the non-edge wells of 384-well microplates, which had drugs dissolved in DMSO (ceftriaxone, levofloxacin, meropenem, rifampicin, tigecycline, trimethoprim, sulfamethoxazole, and cefixime), or 0.1% Triton-X100 in water (cefepime, colistin, and gentamicin), pre-added to the plates using the HP D300E Digital Dispenser. Increasing amounts of single drugs and increasing total amounts of pairs of drugs were used to generate dose–response curves for single drugs and pairs of drugs. For each plate, ≥4 wells were left untreated (no drug added), and 4–8 wells were treated positive controls, which received 3× MIC of one of the drugs tested. These controls were used for calculating the *Z* score, see Data Processing and Quality Control below. Then, 50 µL of sterile media was added to each edge well of the 384-well plates. Plates were grown overnight (18–20 h) with 37°C with shaking. The OD_600_ of each well was measured using a Biotek Synergy HT Microplate Reader. One biological replicate was performed for the dose centering for each species and growth condition, and ≥3 biological replicates were performed for each single drug and pairwise combination tested against each strain and medium (Fig. S2 and S3).

Drug potency measurements are reported as IC_50_ in Fig. S1. The values were log_10_-transformed to generate geometric means and statistical significance. The fold-change in IC_50_ was determined by dividing the geometric means of the IC_50_ values for each condition (antibiotic and strain), by the geometric mean of the lowest IC_50_ within that condition.

### Data processing and quality control

All data analysis was performed in Matlab. The data for each biological replicate were analyzed separately, and log_2_FIC_50_ values and log_2_FIC_90_ for each biological replicate that passed quality control (see below) are reported in Fig. S2 and S3, respectively. Each reported log_2_FIC_50_ value is the arithmetic mean of log_2_FIC_50_ values reported in Fig. S2.

Processing the OD_600_ data by background-subtraction of the median of medium-only edge wells, normalization to the mean of untreated wells in each plate, fitting of the single and pairwise dose–response curves with a three-parameter hill function, and calculation of inhibitory concentration (IC) values based on hill curve parameters was performed as described previously (40). Determination of FIC_50_ scores using the IC_50_ value of the drug pair as well as the IC_50_ values of the component single drugs following the model of Loewe additivity was done as described previously (40). The Ab clinical isolate Ab5075 was highly resistant to gentamicin and meropenem, and the Ab clinical isolate EGA355 was highly resistant to levofloxacin. So, for combinations including gentamicin or meropenem for Ab5075 (gentamicin + meropenem was not tested for Ab5075) and combinations including levofloxacin for EGA355, the drug to which the strain was highly resistant was treated as a sensitizer, and for the combination dose–response curve a constant amount of the sensitizer drug was added to an increasing amount of the other drug in the pair. Instead of calculating the FIC_50_ score for the drug pair, the fold-change between the combination IC_50_ and the non-sensitizer drug IC_50_ was calculated as a measure of potentiation, and in data processing instead of normalizing to the mean of the untreated wells, the wells for the combination dose–response curve were normalized to wells treated with only the sensitizer drug. We consider potentiation analogous to synergy because both involve the combination of two drugs showing greater efficacy than the sum of the drugs’ individual effects. Otherwise, we categorized the drug interaction as “not more effective” if killing appeared similarly to the single doses or “less effective” if more CFU were recovered in the combination dose compared to one or both single doses.

To ensure accuracy and consistency, all biological replicates included in the data set had to pass the following series of quality control criteria. For single-drug dose–response curves, the *R*^2^ of the fitted curve (from which we calculated IC values) had to be ≥0.9, and the 384-well plate on which the dose–response curve was measured had to have a *Z* score of ≥0.4, to ensure sufficient difference between untreated and treated positive control wells requiring consistent growth in the untreated wells and growth inhibition in the positive control wells. The equation we used for *Z* score calculations is $Z=1-\frac{3\times ({\hat{\sigma }}_{p}+{\hat{\sigma }}_{n})}{|{\hat{\mu }}_{p}-{\hat{\mu }}_{n}|}$. In this equation, *${\hat{\mu }}_{n}$* and ${\hat{\mu }}_{p}$ are the average OD_600_ of the untreated and positive control wells, respectively, ${\hat{\sigma }}_{n}$ and ${\hat{\sigma }}_{p}$ and are the standard deviation of the untreated and positive control wells, respectively. We used the same requirements for combination dose–response curves for which FIC_50_ was calculated, with the added criteria that these requirements also had to be met for the component single drugs’ dose–response curves, and the angle score for the combination (a measure of how close the single drugs doses were to achieving equipotency) had to be between 23° and 68° (no more than 22° away from 45°, indicating equipotency and exact measurement along the diagonal).

### Determination of additivity range, synergy, and potentiation

To experimentally determine the window of additivity in our assays, the range of log_2_FIC_50_ scores obtained by measuring three to five drugs from the panel individually in the DiaMOND format with themselves (e.g., a mock combination experiment) against Ab17978, PaO1, and Kp43816 each grown in CAMHB and in M9Glu. For each species in each medium, at least two biological replicate measurements were performed for each drug tested with itself, and the resulting log_2_FIC_50_ scores were used to calculate a 95% CI for additivity for each species in each media. All six of these 95% CI ranges (three species in two media) were within the range of log_2_FIC_50_ = 0.26 and log_2_FIC_50_ = −0.19. Thus, log_2_FIC_50_ scores between −0.19 and 0.26 were considered additive, while scores less than that were considered synergistic and scores greater than that were considered antagonistic.

### Statistical analysis

For each species, we identified the combinations with statistically significant differences in interaction type between growth conditions by performing a two-way ANOVA with multiple comparisons using Tukey’s multiple comparison post-test (*α* = 0.05), with the log_2_FIC_50_ scores from all combinations in CAMHB, M9Glu, and UMM. Combinations were considered statistically significant if *P* ≤ 0.05 in log_2_FIC_50_ between two growth conditions.

For each of the 10 drugs tested, we counted the total number of combinations involving that drug that switched interaction type (e.g., synergy to antagonism) between two growth conditions, across all the growth conditions and species tested. For comparisons between drugs (Fig. 4), we converted each total to a percentage of all the possible switches in interaction type between growth conditions, across all growth conditions and species. Since trimethoprim-sulfamethoxazole and cefixime were only tested in Kp, there are 27 possible switches for each of these two drugs: 1 species × 3 possible media-to-media comparisons × 9 combinations. For the other eight drugs, there are 69 possible switches: 2 species (Ab, Pa) × 3 possible media-to-media comparisons × 7 combinations, plus 1 species (Kp) × 3 possible media-to-media comparisons × 9 combinations (since any of these other eight drugs was also tested with trimethoprim-sulfamethoxazole and cefixime in Kp).

### Mouse infections

For infections, 8- to 12-week-old female or male Swiss Webster mice (Taconic) were anesthetized with isoflurane and infected via the intranasal route with 50 µL containing 10,000 CFU of stationary phase Kp (ATCC43816) grown overnight in L broth and diluted in sterile PBS (45). Prior to infection, mice were weighed to ensure accurate doses of antibiotic(s). Infection was allowed to proceed for 14 h. At this point, stated concentrations of antibiotics diluted in 100 µL of DMSO were administered via intraperitoneal injection. For combination doses, antibiotics were mixed in 100 µL DMSO. A cohort of mice was given 100 µL of DMSO at 14 h post-infection. (Antibiotic concentrations used were based on preliminary experiments that identified antibiotic concentrations that reduced bacterial burden 50- to 500-fold compared to vehicle.) Due to the short half-life of meropenem, a second dose was given at 18 h post-infection. All other antibiotics have longer half-lives in mice (52). Mice were euthanized at 22 h post-infection. Lungs were collected, weighed, and homogenized. Homogenates were diluted, plated on L agar plates, and grown at 37°C overnight. CFUs were counted and used to calculate lung bacterial burden per gram of lung. A two-way ANOVA with Bonferroni’s multiple comparison corrections (*α* = 0.05) was done on log_10_-transformed data to determine statistical significance using GraphPad Prism. All infections were done at least three times with groups of two to four mice/condition and data were compiled. To calculate Bliss interaction scores, log_10_ CFU/g of lung was used to calculate the relative inhibition for each treatment group. These values allowed for the implementation of the Bliss independence model to calculate the expected inhibition if there was no interaction between the two drugs being used (53). To calculate the expected inhibition Eq1 was used, where y_A_ and *y*_*B*_ are the observed fractional growth inhibition by drug A and drug B, respectively, at ½ the dose used for the combination therapy (e.g., 2.5 mg/kg of cefixime and 5 mg/kg of meropenem), *y*_*B*_ is the observed growth inhibition by drug B. Fractional growth inhibition was calculated by log_10_ transforming the geometric means of the CFU/g of lung for each group of mice and dividing the treated groups by the untreated group. The expected growth inhibition is subtracted from the observed growth inhibition to calculate the Bliss score for the combination.


(1) $$Expected\;\;inhibition=\;y_{A}+y_{B}-\left(y_{A}\right)\;\left(y_{B}\right)$$

## SUPPLEMENTAL MATERIAL

Data S1 mbio.00159-24-s0001.xlsx Supporting raw data.

Supplemental Figures and Table mbio.00159-24-s0002.docx Figures S1-S4 and Table S1.

ASM does not own the copyrights to Supplemental Material that may be linked to, or accessed through, an article. The authors have granted ASM a non-exclusive, world-wide license to publish the Supplemental Material files. Please contact the corresponding author directly for reuse.
