# Supplementary material for: Critical role of growth medium for detecting drug interactions in Gram-negative bacteria that model in vivo responses
Source: mBio. 2024 Feb 16;15(3):e00159-24. doi: 10.1128/mbio.00159-24 (PMC10936441; doi:10.1128/mbio.00159-24)
Supplement: Supplemental Figures and Table — Figures S1-S4 and Table S1. [file mbio.00159-24-s0002.docx]

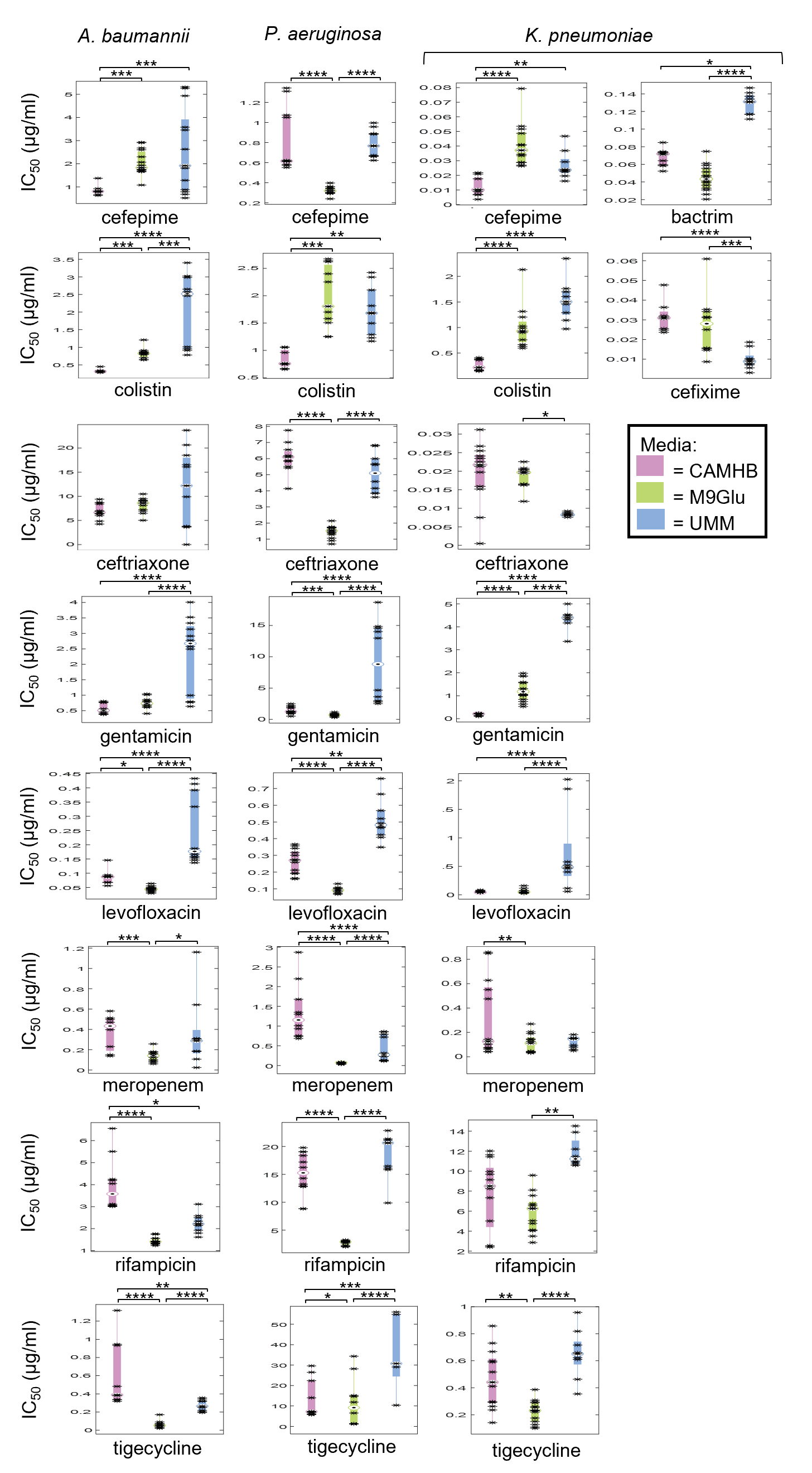


**Figure S1: The effect of media condition on drug potency varies by drug and species identity.** Points on the box-and-whisker plots show the IC_50_ value (μg/ml) for all biological replicates of each drug tested against Ab (left column), Pa (second-from-left column), and Kp (right two columns), in CAMHB (purple boxes), M9Glu (green boxes) and UMM (blue boxes). Outlier replicates were removed using the ROUT method (Q = 2%), and then statistically significant differences between the log10 transformed IC_50_ values of a drug in one growth condition vs. another for each species were calculated using a 2-way ANOVA with multiple comparisons using Tukey’s multiple comparison post-test (α = 0.05). These differences are shown by asterisks above brackets indicating the two growth conditions, with **** indicating adjusted p ≤ 0.0001, *** indicating adjusted p ≤ 0.001, ** indicating adjusted p ≤ 0.01, and * indicating adjusted p ≤ 0.05.


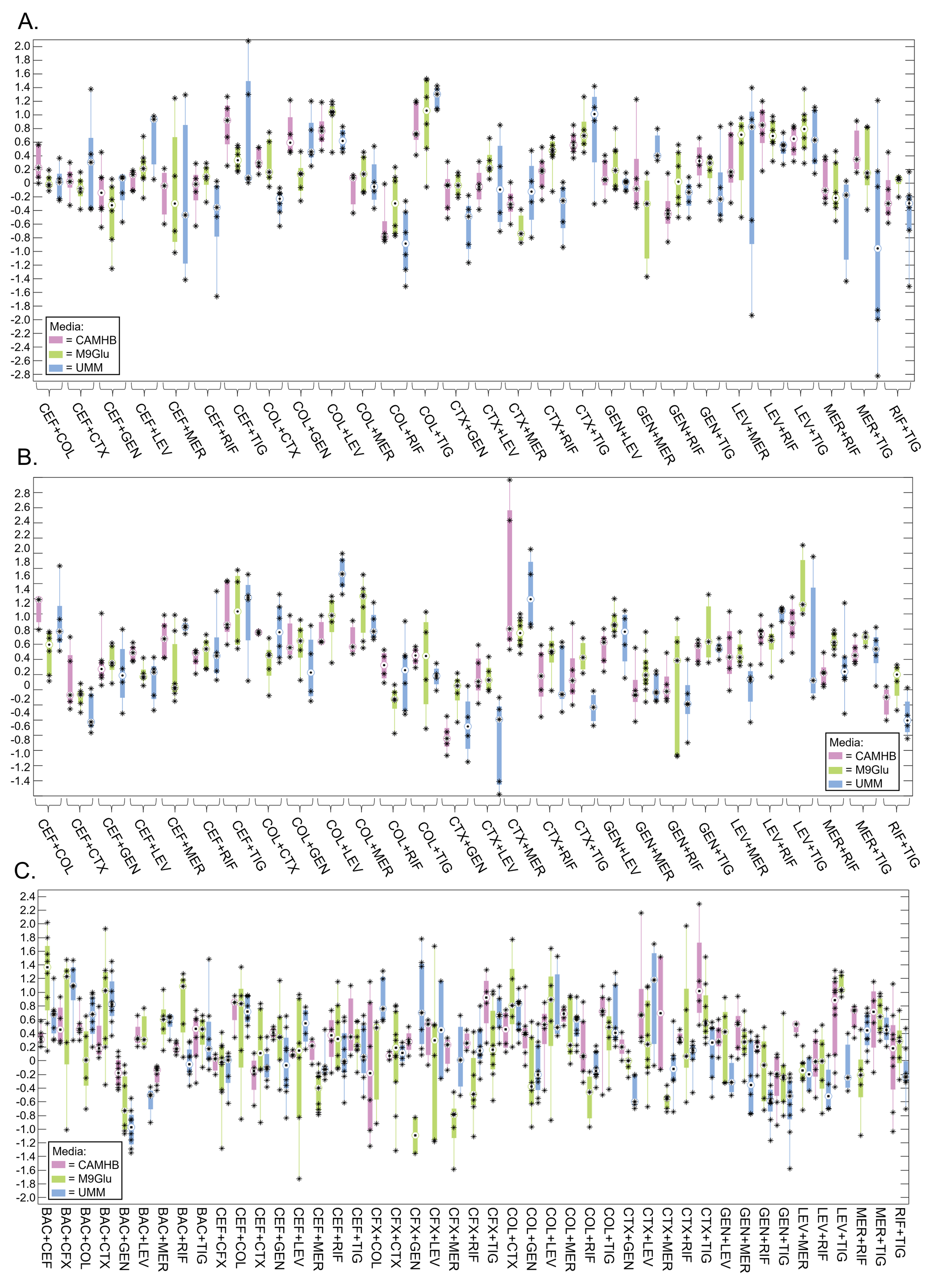


**Figure S2:** Biological replicates of pairwise drug combination log_2_FIC_50_ measurements against (A) *Acinetobacter baumannii* ATCC17978, (B) *Pseudomonas aeruginosa* PaO1, and (C) *Klebsiella pneumoniae* ATCC43816, each grown in CAMHB (purple), M9Glu (green), and UMM (blue). Box plots depict the median (central circle), 25^th^ and 75^th^ percentiles (edges), and whiskers extend to the largest and smallest replicate values. Individual replicate values are marked with a black asterisk.


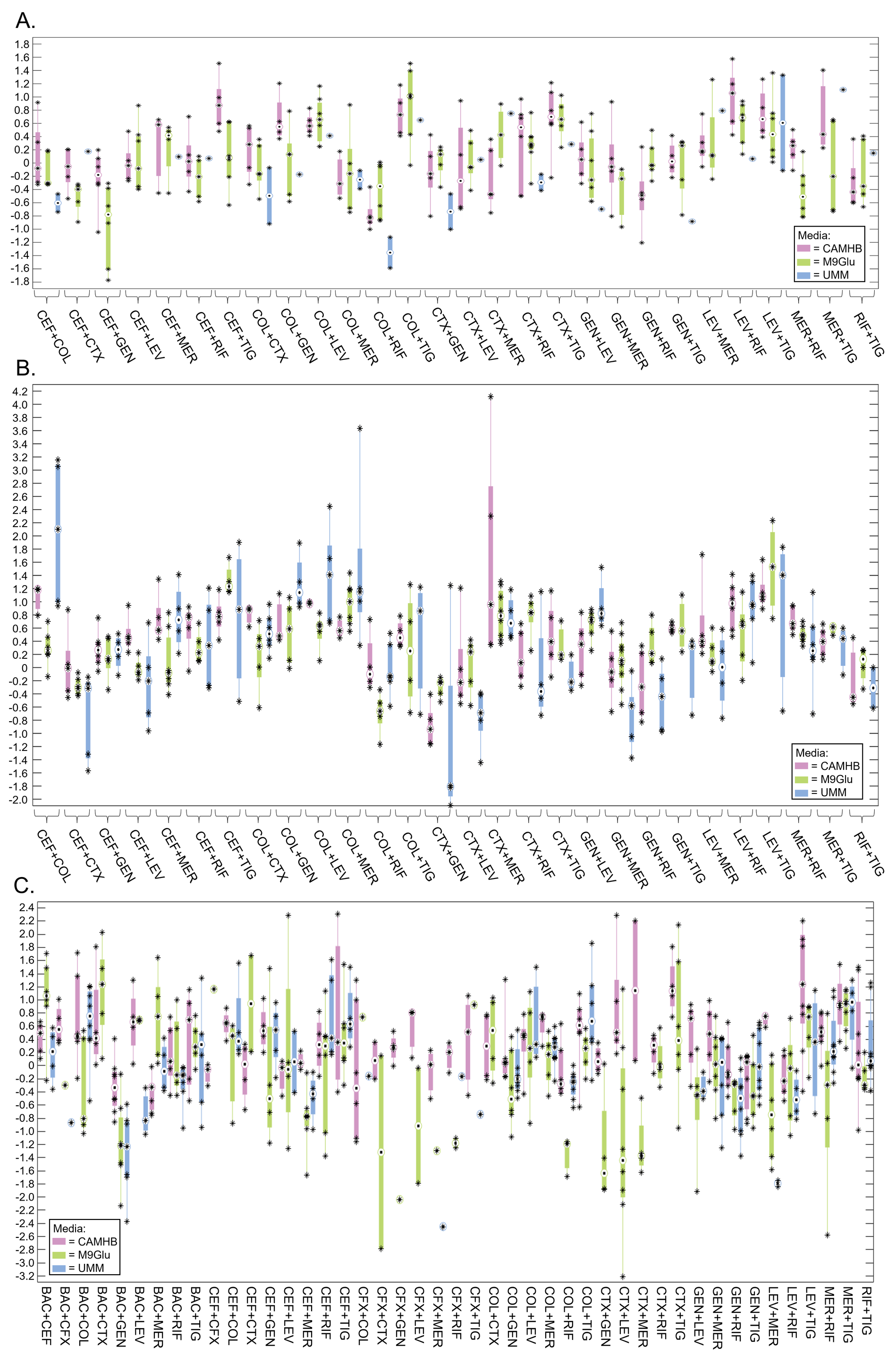


**Figure S3:** Biological replicates of drug combination log_2_FIC_90_ measurements against (A) *Acinetobacter baumannii* ATCC17978, (B) *Pseudomonas aeruginosa* PaO1, and (C) *Klebsiella pneumoniae* ATCC43816, each grown in CAMHB (purple), M9Glu (green), and UMM (blue). Box plots depict the median (central circle), 25^th^ and 75^th^ percentiles (edges), and whiskers extend to the largest and smallest replicate values. Individual replicate values are marked with a black asterisk.


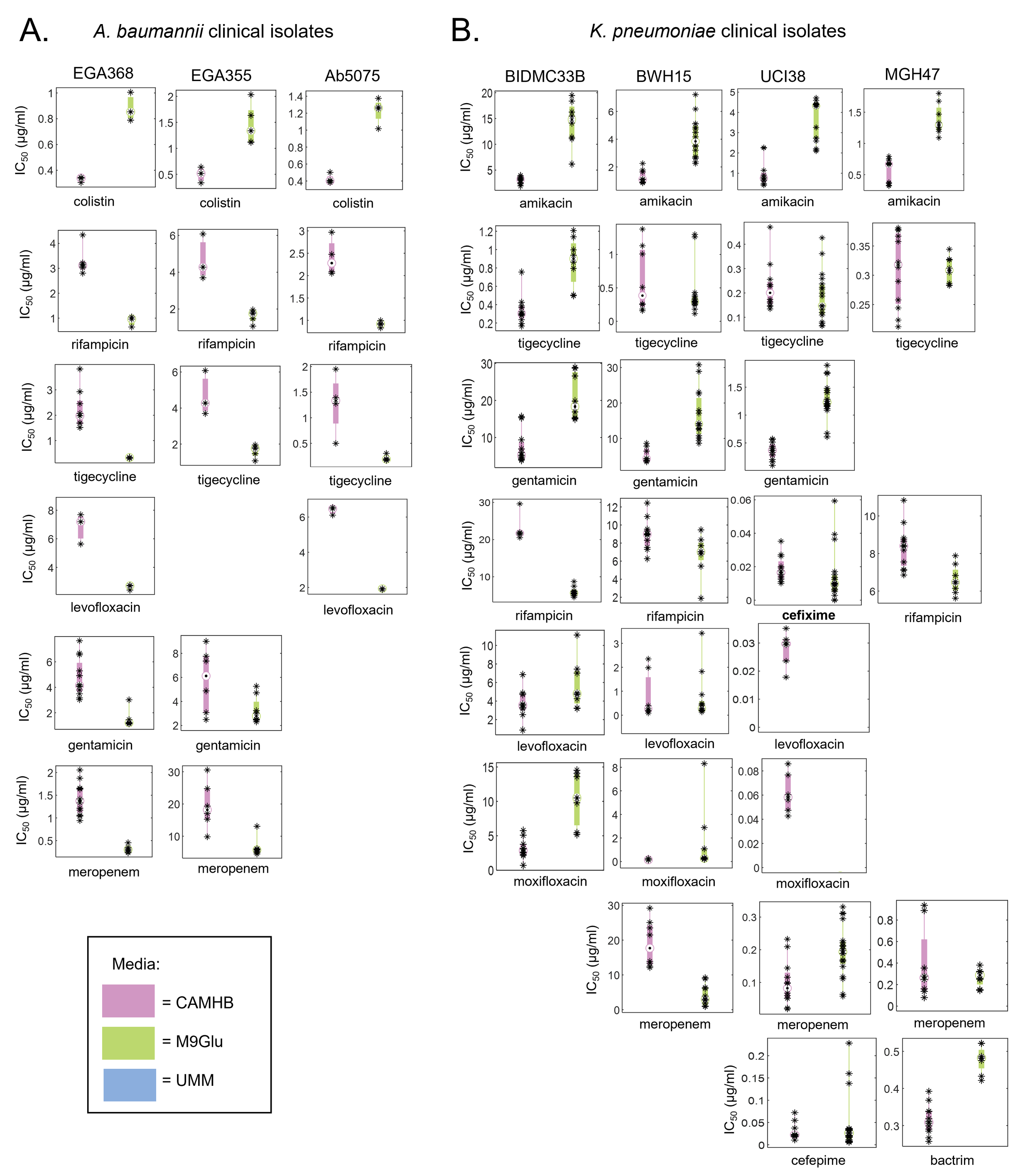


**Figure S4:** Points on the box-and-whisker plots show the IC_50_ value (μg/ml) for all biological replicates of each drug tested against (A) Ab clinical isolates (EGA368, EGA355, Ab5075 and (B) Kp clinical isolates (BIDMC33B, BWH15, UCI38, and MGH47). Replicates in CAMHB are shown in purple boxes, and replicates in M9Glu are shown in green boxes.

**Table S1: Antibiotics used in this study.**

| **Antibiotic** | **Abbreviation** | **Class** | **Mechanism of Action** |
| --- | --- | --- | --- |
| amikacin | AMK | aminoglycoside | protein synthesis inhibition |
| trimethoprim-sulfamethoxazole | BAC | antifolate antibacterial (trimethoprim); sulfonamide (sulfamethoxazole) | folate synthesis inhibition |
| cefepime | CEF | cephalosporin | cell wall synthesis inhibition |
| cefixime | CFX | cephalosporin | cell wall synthesis inhibition |
| colistin | COL | polymyxin | cell membrane disruption |
| ceftriaxone | CTX | cephalosporin | cell wall synthesis inhibition |
| gentamicin | GEN | aminoglycoside | protein synthesis inhibition |
| levofloxacin | LEV | fluoroquinolone | inhibition of DNA replication and transcription |
| meropenem | MER | carbapenem | cell wall synthesis inhibition |
| moxifloxacin | MOX | fluoroquinolone | inhibition of DNA replication and transcription |
| rifampicin | RIF | antimycobacterial | RNA synthesis inhibition |
| tigecycline | TIG | glycylcycline | protein synthesis inhibition |

Class, mechanism of action and 3-letter abbreviation of all antibiotics used in this study.
